# Logged peat swamp forest supports greater macrofungal biodiversity than large‐scale oil palm plantations and smallholdings

**DOI:** 10.1002/ece3.3273

**Published:** 2017-08-02

**Authors:** Siti Noor Shuhada, Sabiha Salim, Frisco Nobilly, Akbar Zubaid, Badrul Azhar

**Affiliations:** ^1^ Department of Forest Production Faculty of Forestry Universiti Putra Malaysia Serdang Selangor Malaysia; ^2^ Department of Animal Science Faculty of Agriculture Universiti Putra Malaysia Serdang Selangor Malaysia; ^3^ Faculty of Science and Technology School of Environmental and Natural Resource Sciences National University of Malaysia Bangi Selangor Malaysia; ^4^ Biodiversity Unit Institute of Bioscience Universiti Putra Malaysia Serdang Selangor Malaysia

**Keywords:** biodiversity, conservation, macrofungi, oil palm, peat swamp forest

## Abstract

Intensive land expansion of commercial oil palm agricultural lands results in reducing the size of peat swamp forests, particularly in Southeast Asia. The effect of this land conversion on macrofungal biodiversity is, however, understudied. We quantified macrofungal biodiversity by identifying mushroom sporocarps throughout four different habitats; logged peat swamp forest, large‐scale oil palm plantation, monoculture, and polyculture smallholdings. We recorded a total of 757 clusters of macrofungi belonging to 127 morphospecies and found that substrates for growing macrofungi were abundant in peat swamp forest; hence, morphospecies richness and macrofungal clusters were significantly greater in logged peat swamp forest than converted oil palm agriculture lands. Environmental factors that influence macrofungi in logged peat swamp forests such as air temperature, humidity, wind speed, soil pH, and soil moisture were different from those in oil palm plantations and smallholdings. We conclude that peat swamp forests are irreplaceable with respect to macrofungal biodiversity. They host much greater macrofungal biodiversity than any of the oil palm agricultural lands. It is imperative that further expansion of oil palm plantation into remaining peat swamp forests should be prohibited in palm oil producing countries. These results imply that macrofungal distribution reflects changes in microclimate between habitats and reduced macrofungal biodiversity may adversely affect decomposition in human‐modified landscapes.

## 1 INTRODUCTION

In the past decades, the expansion of agricultural plantations replacing peatland has occurred at an alarming rate, particularly in poverty‐stricken regions of Southeast Asia. In 1990 to 2010 tropical peat swamp from Malaysia and Sumatra, Indonesia were reduced from 77% to 36%, with only 9% of peatland areas receiving protection (Miettinen, Shi, & Liew, 2012; Posa, Wijedasa, & Corlett, 2011). Despite providing essential services such as soil erosion control, ecosystem stabilization, and carbon storage (Yule, 2010), peat swamp forests have been treated as wastelands (Rijksen & Peerson, 1991). As trees are felled to establish oil palm plantations, the peat swamp forest is drained and resulting decomposition releases substantial carbon emissions into the atmosphere (Murdiyaso, Hergoualc'h, & Verchot, 2010). This is attributed to the fact that cumulative CO_2_ emissions decrease during the high water table conditions, but the emissions may increase during the low water table conditions (Jauhiainen, Takahashi, Heikkinen, Martikainen, & Vasander, 2005). Nowadays, peat swamp forests are being threatened by extensive fire and human exploitations by logging and agricultural industry (Muhammad & Abdullah, 2015). Conversion of forest land cover to agricultural plantations is responsible for causing habitat destruction and loss of forest biodiversity (Rudel, Defries, Asner, & Laurence, 2009; Sodhi & Brook, 2006).

The impact of oil palm plantation expansion on peat swamp. biodiversity is understudied (Posa et al., 2011) which raises numerous concerns. Efforts to understand and conserve peat swamp biodiversity are limited by a lack of information about many taxonomic groups, particularly those groups that are most species rich. Species richness and species abundance are reduced when original land cover changes (Danielsen et al., 2009; Fitzherbert et al., 2008; Foster et al., 2011). These baseline data are mostly available from well‐known taxa such as birds and mammals (Azhar et al., 2011, 2013; Hawa, Azhar, Top, & Zubaid, 2016; Mandal & Shankar Raman, 2016; Prabowo et al., 2016; Sasidhran et al., 2016; Syafiq et al., 2016). There is an urgent need to understand the effects of peat swamp forest conversion to oil palm agriculture on macrofungal diversity. Although macrofungi have important functions in decomposition, nutrient cycling, and nutrient uptake, little is known about macrofungal diversity, as it is ephemeral and has enigmatic growing patterns which make identification difficult (Schmit et al., 2005).

Macrofungi are sensitive to habitat modification (Brown, Bhagwat, & Watkinson, 2006; Halme et al., 2013). Previous studies have found that macrofungal diversity would be affected directly in countries experiencing modification in land cover (Kasel, Bennett, & Tibbits, 2008; López‐Quintero, Straastsma, Franco‐Molano, & Boekhout, 2012; Paz, Gallon, Putzke, & Ganade, 2015), especially when the floristic composition and structural characteristics are altered (Brown et al., 2006; Gómez‐Hernández & Williams‐Linera, 2011). The conversion of native forests to exotic crop plantations has been found to lower the number of macrofungal decomposer species, most likely due to changes in substrate availability and quality (Heilmann‐Clausen et al., 2014; Paz et al., 2015). Despite their functional importance, knowledge of macrofungal diversity is still lacking in both peat swamp forest and oil palm production landscapes due to the paucity of information about these taxa. The data sets for macrofungal diversity in major geographical regions of the world are incomplete, and thus, the existing numbers of macrofungal species represent very conservative estimates for macrofungal diversity in each region (Lodge, 1997; Lodge & Cantrell, 1995; Mueller & Schmit, 2007; Piepenbring, 2007). Lacking data on macrofungal diversity with visible fruiting bodies in oil palm plantation is one of the largest knowledge gaps for fungi in peatlands. Thus, detailed studies of the effect of peat swamp and forest conversion to oil palm on macrofungal communities is a priority for conservation research.

In 2008, forest fires occurred extensively in Malaysia, degrading the forest area particularly in the North Selangor Peat Swamp Forest (NSPSF), and ultimately resulting in 1,231 ha of the country's second largest peat swamp forest being converted into an oil palm plantation (Yule & Gomez, 2008). This means that once this area became degraded, it was easier for oil palm to expand and use the area. Encouraged by strong global market demand in oil palm products, large‐scale plantations and smallholdings currently surround at least 60% of the NSPSF perimeter, and more NSPSF land is scheduled for clearance to make way for plantations (Azhar et al., 2011).

**Figure 1:**
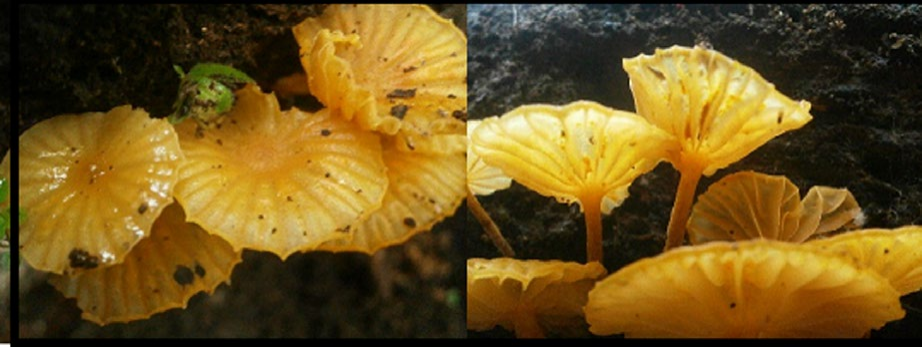
Fruiting bodies of *Lichenomphalia* sp. are commonly encountered in peat swamp forest, but are absent in oil palm cultivation areas

Our study provides new information related to the biodiversity patterns of macrofungal diversity in human‐modified peat soil habitats (Figure 1). This baseline information is essential to formulate successful conservation strategies (Brown et al., 2006; Lindenmayer et al., 2012). First, we compared morphospecies richness and number of macrofungal clusters between logged peat swamp forest, oil palm plantation (>50 ha; private business), and smallholdings (<50 ha; independent farmer) including those that were either monoculture or polyculture system. Secondly, we contrasted vegetation structure and substrate availability between the four habitat types. Thirdly, we determined key environmental and vegetation structure attributes which influence macrofungal diversity.

## 2 MATERIALS AND METHODS

### 2.1 Study area description

We conducted this study at the North Selangor Peat Swamp Forest (NSPSF) and nearby oil palm planted areas. Surveys were conducted from November 2015 to January 2016 during the Northeast and Southwest monsoon season. The mean temperature of the monsoon months in the study area is 31°C; rainfall is between 200 and 500 mm annually (MMD (Malaysian Meteorological Department), 2015).

The NSPSF (N 3°40′26.56″, E 101°4′29.52″) is located at the north western part of Selangor with an elevation of 16 m above sea level. The NSPSF is a secondary forest embracing an area of 73,593 ha where 95% of the area is logged peat swamp forest and 5% is dipterocarp forest (Azhar et al., 2011, 2013). NSPSF consists of three forest reserves; Raja Musa Forest Reserve (23,486 ha), Sungai Karang Forest Reserve (50,106 ha), and Sungai Dusun Wildlife Reserve (4,330 ha) (Parish et al., 2014). Currently, the NSPSF is being threatened by forest fire and oil palm plantation expansion (Azhar et al., 2011; Sasidhran et al., 2016). Despite being designated as forest reserve, more than 1,000 ha of NSPSF has been cleared for plantation purposes (Sasidhran et al., 2016). Sungai Dusun Wildlife Reserve is the only hope for biodiversity conservation in the area as it has been formally appointed as a protected area (Adila et al., 2017; Sasidhran et al., 2016).

**Figure 2:**
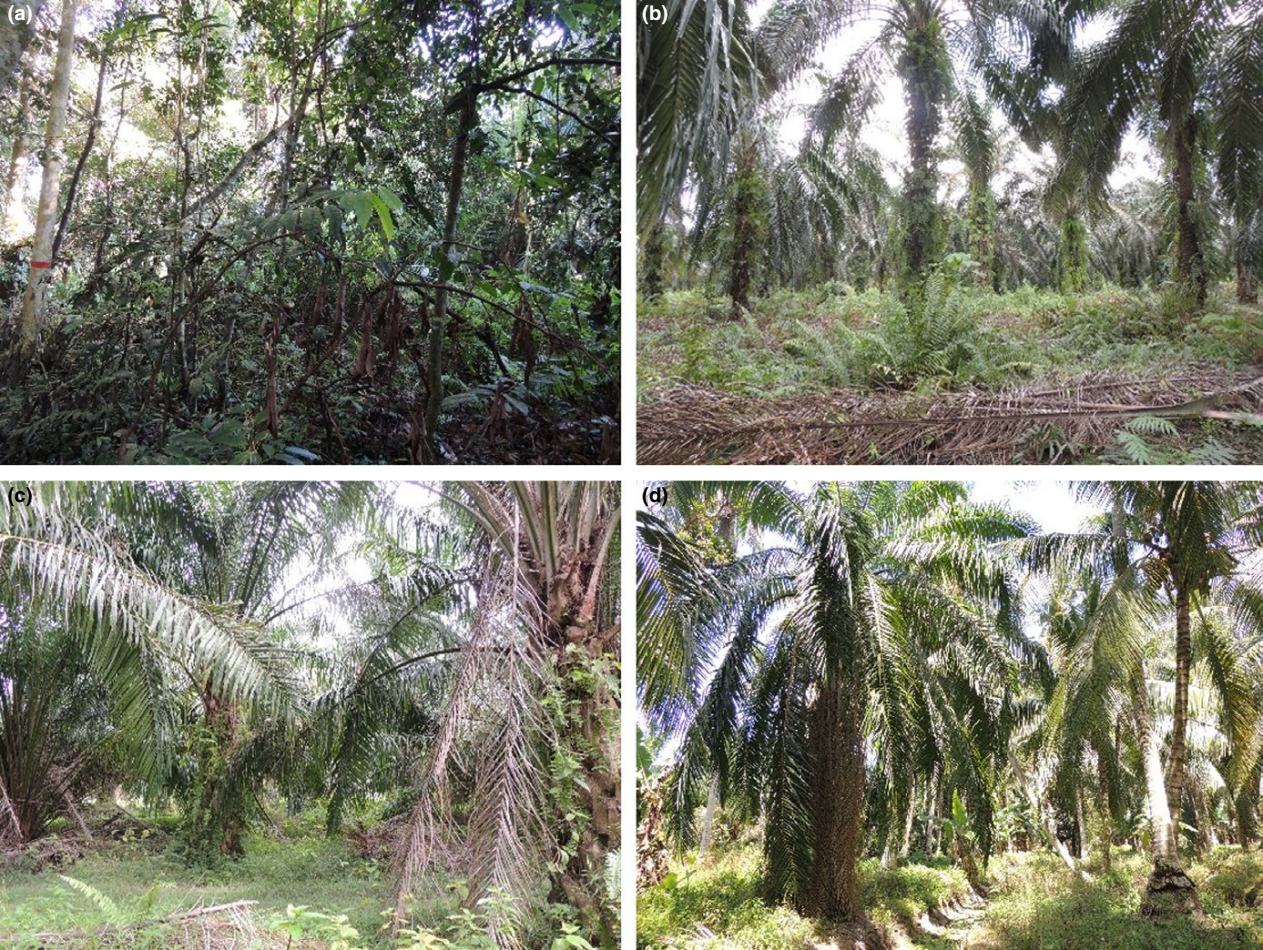
Type of peatland habitats surveyed (a) natural peat swamp forest, (b) large‐scale oil palm plantation, (c) oil palm monoculture smallholding, and (d) oil palm polyculture smallholding

Agricultural plantations surveyed in this study were originally peat land but are now planted with oil palm (*Elaeis guineensis*), aged 8‐years at the time of sampling. Large‐scale plantations, covering an area of 2,000 ha, were managed by a large corporation with the use of advanced machinery (Azhar et al., 2011). Smallholdings, defined as semi‐traditional cultivation area of less than 50 ha, were managed by small‐scale farmers and were less dependent on modern infrastructure (Azhar et al., 2015). Two different smallholder landscapes were studied: monoculture and polyculture. Monoculture smallholdings were planted mainly with oil palms with no intercropping. Polyculture smallholdings, on the other hand, often practiced intercropping, where oil palm plants were planted side by side with subsidiary commercial crops such as banana, coconut, cassava, coffee, pineapple, mangoes, jackfruit, and tapioca (Figure 2).

### 2.2 Sampling design

**Figure 3:**
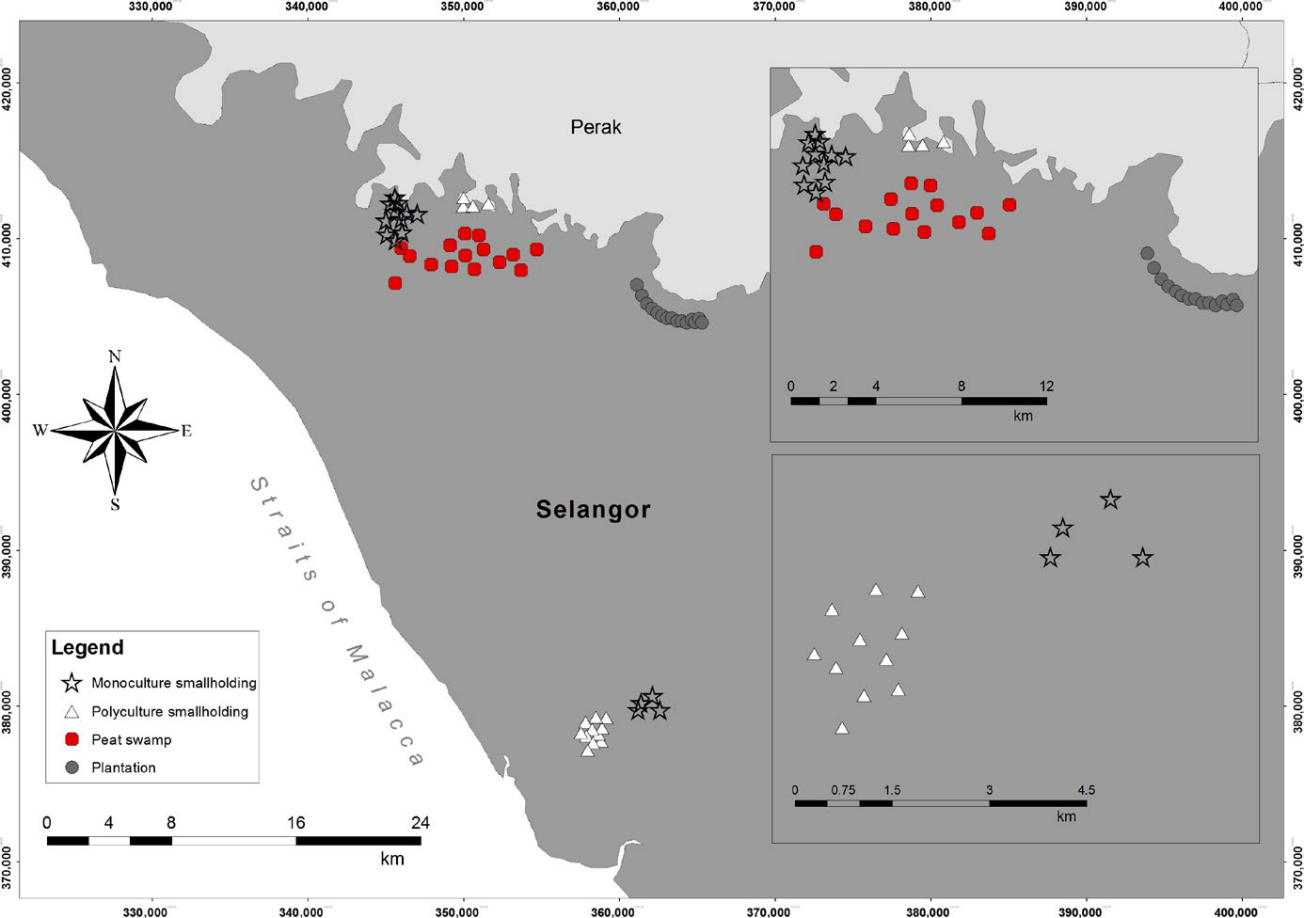
Map of study areas showing sampling plots in peat swamp forest, large‐scale oil palm plantation, monoculture smallholding, and polyculture smallholding

We used systematic sampling with a random starting point (Morrison, Block, Strickland, Collier, & Peterson, 2008). First plot was randomly established in different habitats (at least 100 m from edge) such as large‐scale oil palm plantation, monoculture and polyculture smallholdings, and peat swamp forest. Sixty circular plots were surveyed in the four habitats. Within each of the treatments (i.e., type of habitat), 15 circular plots of 20 m radius were established. To ensure no overlapping occurred, plots were distanced at least 250 m apart. The geographical coordinates of each sampling plot were determined using a geographic positioning device (GPS II Plus, Garmin Ltd., Olathe, Kansas) (Figure 3). Sampling of macrofungi was conducted from 0930 to 1130.

### 2.3 Macrofungal sampling

A team comprising five people conducted direct searches within each sampling plot. The team spent 20–30 min at each sampling plot to search for macrofungal specimens.

To increase the chances of encountering macrofungi, each sampling plot was visited after a rainy day because mushroom fruiting bodies are likely to appear in the most humid conditions (Henkel, Meszaros, Aime, & Kennedy, 2005; López‐Quintero et al., 2012). Throughout this study, macrofungi were identified following Lodge et al. 2004, Mueller et al. (2004), and López‐Quintero et al. (2012).

We concentrated our sampling on visible basidiomycetes and ascomycetes sporocarps detected on the forest floor, fallen logs, as well as on living and dead standing trees (Paz et al., 2015). At each sampling plot, we recorded the number of macrofungal clusters. A cluster was recorded as one observation irrespective of the number of sporocarps in that cluster (Brown et al., 2006). Voucher specimens were also collected and stored in a multiple partition plastic box for ex situ identification. Sporocarp surveys represent a cost‐effective and reliable method to survey macrofungi, because they concentrate on the reproductive parts of the fungal species (Halme, Heilmann‐Clausen, Rämä, Kosonen, & Kunttu, 2012; Paz et al., 2015).

### 2.4 Macrofungal classification and identification

The identification of macrofungi was conducted using published keys (Kirk, Cannon, Minter, & Stalpers, 2008). Each species was identified to genus and family level based on morphological features, such as color, shape, size, and surface texture (Mueller et al., 2004). We recorded the morphological details such as pileus/cap (diameter, texture, margin, shape); lamellae/gills (arrangement, attachment, color); stipe (height, diameter, shape, texture, color); and substrate attachment to help on the taxonomic identification (Lee, Alias, Jones, Zainuddin, & Chan, 2012; Paz et al., 2015; Pereira, 1984). Collected sporocarps were then dried to prevent rotting and damage. Agarics (gill fungi) were dried around 3–8 hr at 45°C. The polyporales (bracket fungi) were dried to 24–48 hr at 55°C using a food dehydrator (Himmel food dehydrator V2 series, Nature Himmel Marketing Sdn Bhd, Selangor, Malaysia).

### 2.5 Measurement of environmental variables and vegetation structure characteristics

At each sampling plot, environmental variables comprising air temperature, humidity, soil pH, soil moisture, and wind speed were recorded (Table 1). Air temperature and relative humidity were measured with a mini environmental quality meter (Sper Scientific 850070, Sper Scientific Ltd., Scottsdale, United States) at 1 m above ground. We recorded soil moisture and pH using a soil pH and moisture tester (DM‐15, Takemura Electric Works Ltd., Tokyo, Japan) at a depth of about 7 cm. We measured canopy cover by photographing the canopy and digitally analyzing the images. The photographs of tree canopy cover for each plot were captured at about 0.5 m from the ground using a DSLR camera, and canopy cover and closure were estimated using Canopy Analysis (Korhonen & Heikkinen, 2009) in MATLAB software (Mathworks, Natick, Massachusetts, USA). We recorded the substrate macrofungi were growing on at each sampling plot. This included the following: branch (diameter >2.5 cm), twig (diameter <2.5 cm), fallen leaves, fruit shells, living tree, trunk, and soil (Gibertoni, Santos, & Cavalcanti, 2007; López‐Quintero et al., 2012).

**Table 1:** Summary statistics of environmental, vegetative structure, and substrate availability in logged peat swamp forest, large‐scale oil palm plantation, monoculture, and polyculture smallholdings

Variable	Habitat	Mean	*SD*	Minimum	Median	Maximum
Air temperature (°C)	Forest	29.01	1.091	27.1	29.2	30.8
	Large‐scale	30.55	1.482	28.3	30.6	32.6
	Monoculture	32.53	0.565	31.4	32.7	33
	Polyculture	32.51	1.720	28.4	33	34.2
Humidity (%)	Forest	85.93	2.519	82.1	86.1	90.9
	Large‐scale	73.21	9.783	59	77.3	84.3
	Monoculture	68.99	3.219	66	67.7	73.7
	Polyculture	60.83	8.187	51.6	56.9	77.7
Soil moisture (%)	Forest	6.3	0.727	5	6	7
	Large‐scale	4.347	0.932	3.3	4	6
	Monoculture	3.547	1.030	2	3	4.7
	Polyculture	3.133	0.946	2	3	5
Soil pH	Forest	4.353	0.559	3.2	4.2	5
	Large‐scale	5.193	0.576	4.5	5	6.4
	Monoculture	5.773	0.435	5.3	6	6.6
	Polyculture	5.647	0.487	5	5.4	6.5
Wind speed (m/s)	Forest	0	0	0	0	0
	Large‐scale	0.033	0.0488	0	0	0.1
	Monoculture	0.1	0.131	0	0.1	0.4
	Polyculture	0.107	0.103	0	0.2	0.2
Canopy closure (%)	Forest	90.067	2.809	84.5	90	95
	Large‐scale	71.067	13.635	48.5	75.5	91
	Monoculture	70.667	12.953	43	73.5	82.5
	Polyculture	72.5	14.537	32	78	87.5
Canopy cover (%)	Forest	98.133	1.747	94	99	100
	Large‐scale	82	12.791	62.5	83	99
	Monoculture	79.367	13.364	51	81.5	91
	Polyculture	81.633	15.732	37	89	94.5
Substrate	Forest	5.8	1.656	2	6	8
	Large‐scale	2.267	1.099	1	2	4
	Monoculture	2.4	0.986	1	2	4
	Polyculture	3.067	1.335	1	3	5

### 2.6 Statistical analysis

We contrasted the observed morphospecies richness with the Chao 1 bias correction estimator for the species richness in EstimateS version 9.1 to assess the overall sampling effort (Colwell, Mao, & Chang, 2004). We used ACE (Abundance Coverage‐based Estimator) to take into account imperfect detection of rare species (Colwell & Coddington, 1994).

We performed one‐way ANOVA to compare the morphospecies richness, number of macrofungal clusters, and environmental variables between habitats. Normality tests (i.e., Shapiro‐Wilk) were run to check the data distribution. Data related to species richness, number of macrofungal cluster, and substrates were square root transformed. Microclimate and vegetative structure attributes (e.g., air temperature, humidity, wind speed, soil moisture, canopy cover, and canopy closure) were log‐transformed. The soil pH on the other hand was not transformed as it is already normally distributed. Tukey's post hoc tests were used to compare between different habitats where significant differences were detected. All univariate analyses were conducted in Genstat version 15 software (VSNI, Hemel, Hempstead, UK).

To determine the contribution of dominant morphospecies to the macrofungal community, we performed one‐way analysis of similarity percentage (SIMPER). Prior to the analysis, the number of morphospecies count data was square root transformed. Bray‐Curtis index was performed to calculate the compositional dissimilarity between different habitats. We used a nonmetric multidimensional scaling (NMDS) to visualize difference in species composition between habitat types. We used PRIMER version 6 (PRIMER‐E Ltd, Plymouth) to perform all multivariate analyses.

We examined the spatial autocorrelation in residuals by calculating Global Moran's Index in the ArcGIS™ version 10.1 (ESRI). The *p* value was used to reject or accept the null hypothesis which states that the analyzed attribute is randomly distributed among the features in the study area (Mitchell, 2005). We used inverse distance (nearby neighboring features have a larger influence on the computations for a target feature than features that are far away) to calculate Global Moran's Index.

## 3 RESULTS

A total of 757 macrofungal clusters were collected, representing 127 morphospecies where 43.07% (*n* = 326 clusters) clusters were recorded from logged peat swamp forest. We recorded 19.68% (*n* = 149 clusters) in large‐scale oil palm plantation. Monoculture and polyculture smallholdings had 15.59% (*n* = 118 clusters) and 21.66% (*n* = 164 clusters), respectively. With respect to the sampling completeness, the sampling effort in logged peat swamp forest was compared with the Chao 1 and ACE estimators, yielding 64% and 71% of the “true” species richness for macrofungi, respectively. In the plantation estate, the sampling effort yielded 46% (against ACE) and 65% (against Chao 1) of the “true” species richness. In monoculture smallholding, the sampling effort resulted in 71% (against ACE) and 84% (against Chao 1) of the “true” species richness. Similarly, the sampling effort in polyculture smallholdings produced 70% (against ACE) and 82% (against Chao 1) of the “true” species richness.

### 3.1 Patterns of macrofungal cluster and morphospecies

**Figure 4:**
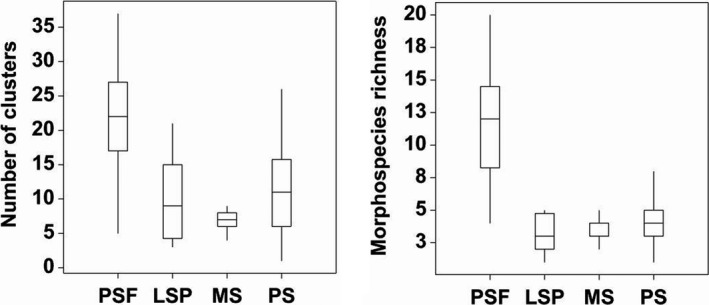
Boxplots showing number of macrofungal clusters and morphospecies richness at four type of peatland habitats. PSF, LSP, MS, and PS denote peat swamp forest, large‐scale plantation, monoculture smallholding, and polyculture smallholding, respectively. The bottom and top of the box are the first and third quartiles, and the band inside the box is the median (the second quartile). The whiskers represent the extreme values (the locations of the minimum and maximum)

To test our first hypothesis, we compared macrofungal cluster assemblages and morphospecies richness between logged peat swamp forest, large‐scale plantation, monoculture, and polyculture smallholdings. Based on analysis of variance (ANOVA), we found that the number of macrofungal clusters was significantly greater (*F*_3,56_ = 9.96; *p *<* *.001) in logged peat swamp forest ($\overset{¯}{x}$ ± *SE* = 21.73 ± 2.427 clusters) than in large‐scale plantation ($\overset{¯}{x}$ ± *SE* = 9.933 ± 1.736 clusters), monoculture ($\overset{¯}{x}$ ± *SE* = 7.867 ± 0.990 clusters), and polyculture smallholdings ($\overset{¯}{x}$ ± *SE* = 10.93 ± 1.744 clusters; Figure 4). A post hoc Tukey test showed that no significant difference in the number of macrofungal clusters between large scale plantations, monoculture, and polyculture smallholdings.

Our result revealed that the logged peat swamp forest ($\overset{¯}{x}$ ± *SE* = 11.73 ± 1.173 morphospecies) supported significantly greater morphospecies richness of macrofungi (*F*_3,56_ = 36.99; *p *<* *.001) compared to the large‐scale plantation ($\overset{¯}{x}$ ± *SE* = 3.267 ± 0.371 morphospecies), monoculture ($\overset{¯}{x}$ ± *SE* = 3.533 ± 0.236 morphospecies), and polyculture smallholdings ($\overset{¯}{x}$ ± *SE* = 4.267 ± 0.431 morphospecies; Figure 4). A total of 61, 21, 21, and 24 morphospecies were collected from peat swamp forest, large‐scale plantation, monoculture, and polyculture smallholdings, respectively. All other comparisons of morphospecies richness based on a post hoc Tukey test were not significant.

We found that the spatial distribution of residuals was the result of random spatial process (peat swamp Moran's Index = −0.277; z‐score = −1.237; *p* = .216; large‐scale plantation Moran's Index = 0.029; z‐score = 0.535; *p* = .593; *p* = .216; monoculture smallholding Moran's Index = −0.126; z‐score = −0.279; *p *=* *.780; *p *=* *.216; polyculture smallholding Moran's Index = −0.267; z‐score = −1.328; *p* = .184).

### 3.2 Vegetative structure and substrate availability

**Figure 5:**
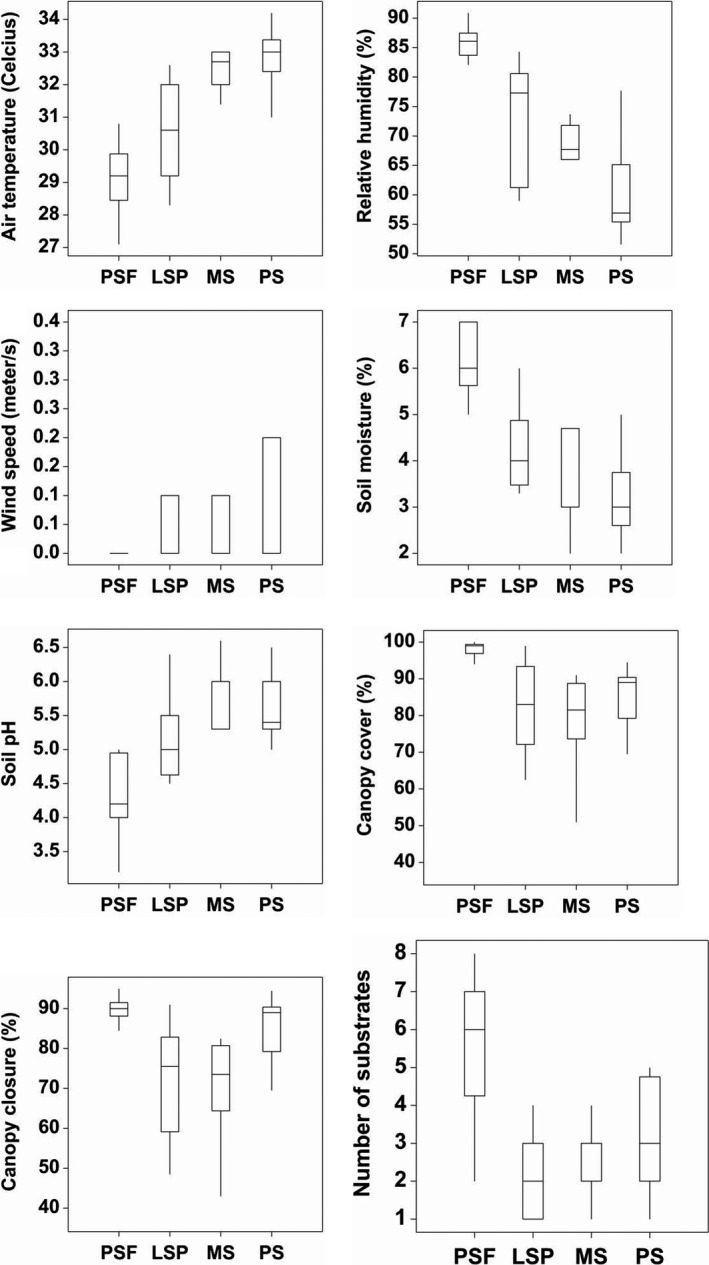
Boxplots showing environmental variables across four different habitat types. PSF, LSP, MS, and PS denote peat swamp forest, large‐scale plantation, monoculture smallholding, and polyculture smallholding, respectively. The bottom and top of the box are the first and third quartiles, and the band inside the box is the median (the second quartile). The whiskers represent the extreme values (the locations of the minimum and maximum)

We found that the canopy cover was significantly denser (*F*_3,56_ = 5.36; *p* = .003) in peat swamp forests ($\overset{¯}{x}$ ± *SE* = 98.133 ± 0.451%; Figure 5) compared to large‐scale plantations ($\overset{¯}{x}$ ± *SE* = 82.000 ± 3.303%), monoculture ($\overset{¯}{x}$ ± *SE* = 79.367 ± 0.237%), and polyculture smallholdings ($\overset{¯}{x}$ ± *SE* = 81.633 ± 4.062%). A post hoc Tukey test showed that all other comparisons of canopy cover were not significant. In terms of canopy closure, we also found significantly denser canopy (*F*_3,56_ = 6.22; *p* = .001) in peat swamp forest ($\overset{¯}{x}$ ± *SE* = 90.067 ± 0.725%; Figure 5) than large‐scale plantation ($\overset{¯}{x}$ ± *SE* = 71.067 ± 3.521%), monoculture ($\overset{¯}{x}$ ± *SE* = 70.667 ± 3.344%), and polyculture smallholdings ($\overset{¯}{x}$ ± *SE* = 72.500 ± 3.753%). Similar to canopy cover, all other comparisons of canopy closure based on a post hoc Tukey test were not significant.

We found that the substrate availability was significantly greater (*F*_3,56_ = 18.96; *p *<* *.001) in peat swamp forest ($\overset{¯}{x}$ ± *SE* = 5.800 ± 0.428; Figure 5) than large‐scale plantation ($\overset{¯}{x}$ ± *SE* = 2.267 ± 0.284), monoculture ($\overset{¯}{x}$ ± *SE* = 2.400 ± 0.254), and polyculture smallholding ($\overset{¯}{x}$ ± *SE* = 3.067 ± 0.345). A post hoc Tukey test showed that all other comparisons of substrate availability were significant. Of 14 different substrate types, 11 records, that is, coarse wood, twig, living tree, stump, fallen tree, trunk, branch, buttress, fruit, leaf litter, and soil are observed from peat swamp forest. Highest production (120 collections) of sporocarps from forest macrofungal was observed on coarse wood. Substrate availability in both large‐scale plantation and monoculture smallholding was limited to oil palm fronds.

### 3.3 Microclimate variables

Our results also show that there were significant differences in air temperature (*F*_3,56_ = 25.48; *p *< .001), humidity (*F*_3,56_ = 32.91; *p* < .001), soil moisture (*F*_3,56_ = 26.88; *p *<* *.001), soil pH (*F*_3,56_ = 23.12; *p *<* *.001), and wind speed (*F*_2,19_ = 4.94; *p *<* *.019) between different habitats (Figure 5). We found that air temperature in peat swamp forests was significantly lower ($\overset{¯}{x}$ ± *SE* = 29.01 ± 0.282°C) than in large‐scale plantations ($\overset{¯}{x}$ ± *SE* = 30.55 ± 0.383°C), monoculture ($\overset{¯}{x}$ ± *SE* = 32.53 ± 0.146°C), and polyculture smallholdings ($\overset{¯}{x}$ ± *SE* = 32.51 ± 0.444°C; Figure 5). A post hoc Tukey test showed that large‐scale plantations differed significantly in terms of air temperature from monoculture and polyculture smallholdings. No significant difference in air temperature was detected between monoculture and polyculture smallholdings.

As for humidity, the peat swamp forest records were significantly higher ($\overset{¯}{x}$ ± *SE* = 85.93 ± 0.650%) than in large‐scale plantations ($\overset{¯}{x}$ ± *SE* = 73.21 ± 2.526%), monoculture ($\overset{¯}{x}$ ± *SE* = 68.99 ± 0.831%), and polyculture smallholdings ($\overset{¯}{x}$ ± *SE* = 60.83 ± 2.114%; Figure 5). Further analysis showed that polyculture smallholdings differed significantly in terms of relative humidity from monoculture smallholdings and large‐scale plantations. No significant difference in relative humidity was detected between smallholdings and large‐scale plantations.

In terms of soil moisture, the peat swamp forest records were significantly higher ($\overset{¯}{x}$ ±* SE* = 6.3 ± 0.188) than in large‐scale plantations ($\overset{¯}{x}$ ± *SE* = 4.347 ± 0.241), monoculture ($\overset{¯}{x}$ ± *SE* = 3.547 ± 1.061), and polyculture smallholdings ($\overset{¯}{x}$ ± *SE* = 3.133 ± 0.244; Figure 5). Based post hoc Tukey test, all other comparisons of soil moisture were significant. We also found much more acidic soil in peat swamp forest ($\overset{¯}{x}$ ± *SE* = 4.353 ± 0.144) compared to large‐scale plantations ($\overset{¯}{x}$ ± *SE* = 5.193 ± 0.149), monoculture ($\overset{¯}{x}$ ± *SE* = 5.773 ± 0.112), and polyculture smallholdings ($\overset{¯}{x}$ ± *SE* = 5.647 ± 0.237; Figure 5). A post hoc Tukey test showed that all other comparisons of soil pH were significant. Lastly, we recorded a wind speed of zero at the peat swamp forest. However, wind speed was recorded in polyculture smallholdings ($\overset{¯}{x}$ ± *SE* = 0.107 ± 0.0267 ms^−1^), large‐scale plantations ($\overset{¯}{x}$ ± *SE* = 0.0333 ± 0.0126 ms^−1^), and monoculture smallholdings ($\overset{¯}{x}$ ± *SE* = 0.1 ± 0.0338 ms^−1^; Figure 5), but the comparisons were not significant.

### 3.4 Macrofungal community

**Figure 6:**
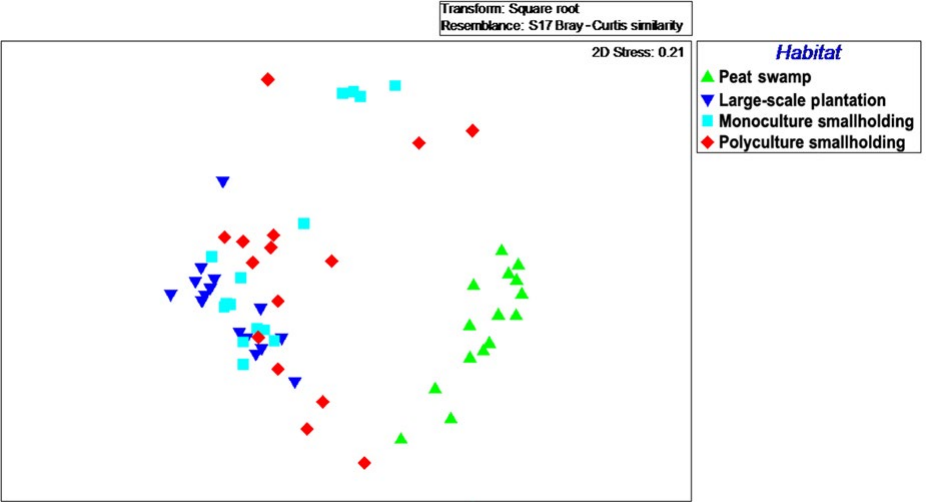
Nonmetric multidimensional scaling (NMDS) ordination comparing the macrofungal community between four different peatland habitats including those converted into oil palm agricultural areas

The macrofungal community differed significantly between the peat swamp forest and all other habitat types (ANOSIM, *R*_global_ = 0.457; Number of permutations: 999; *p* < .001; Table 2). All three oil palm plantation habitats contained species that are not typical of peat swamp forest (Figure 6).

**Table 2:** Analysis of similarities (ANOSIM) in species composition of macrofungi between natural peat swamp (PSF, *n* = 15), large‐scale oil palm plantation (LSP, *n* = 15), monoculture smallholding (MS, *n* = 15), and polyculture smallholding (PS, *n* = 15)

Comparisons	*R*‐value	*p*‐value
PSF, LSP	0.948	.001
PSF, MS	0.732	.001
PSF, PS	0.726	.001
LSP, MS	0.136	.005
LSP, PS	0.377	.001
MS, PS	0.048	.091

**Figure 7:**
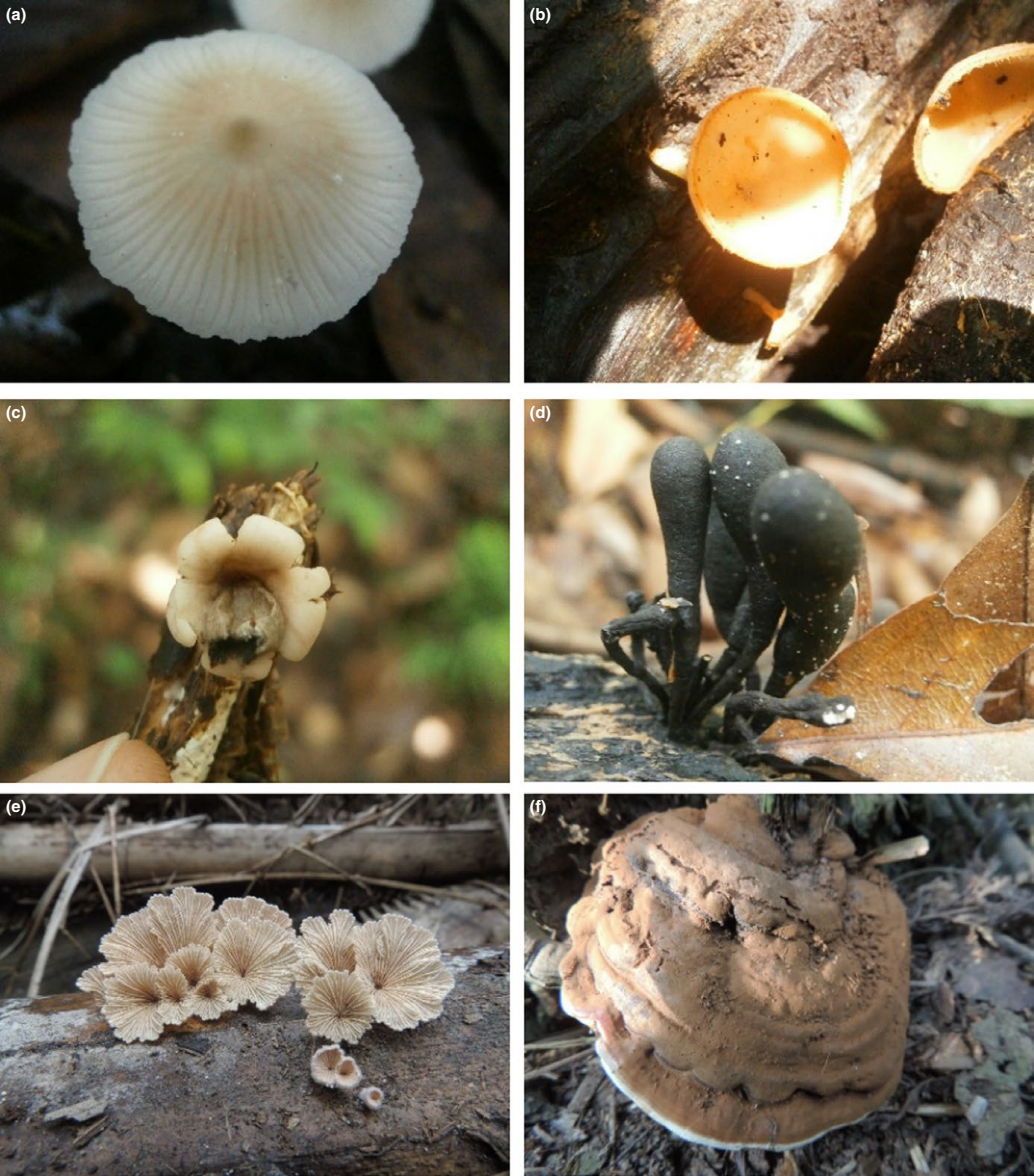
Photographs of macrofungal morphospecies from the NSPSF and oil palm cultivation areas: (a) *Inocybe* sp. found on wood; (b) *Cookeina sulcipes* growing on wood; (c) mature *Geastrum javanicum*; (d) *Xylaria* sp. found on fallen tree; (e) *Schizophyllum commune* growing on oil palm frond; and (f) *Ganoderma* sp. found on oil palm buttress at polyculture smallholding

One‐way similarity percentages (SIMPER) was conducted to study the morphospecies richness contribution in all habitats. From the analysis, a total of 13 morphospecies made up 90.04% (Table 3) of the total macrofungal assemblage in the peat swamp forest. The peat swamp forest community predominately consisted of basidiomycetes and ascomycetes, from the families Polyporaceae, Marasmiaceae, and Mycenaceae. The most common species was *Inocybe* sp. (Figure 7a), recorded in 13 of 15 sampling units, and followed by *Marasmius* sp., and *Mycena* sp., which were commonly found on forest litter and soil. Other common macrofungal morphospecies included *Cookeina* sp. (Figure 7b), *Geastrum javanicum* (Figure 7c), and *Xylaria* sp. (Figure 7d). Besides these, uncommon species, such as *Hericium cirrhatum*, were found on decaying trunks. Within the large plantations and monoculture and polyculture smallholdings, *Schizophyllum commune* (Figure 7e) was found in almost all sampling units, mainly on oil palm fronds. Although *S. commune* was the commonest species in large‐scale and monoculture smallholding, *Ganoderma* sp. (Figure 7) was the commonest in polyculture plantations, constituting 31.20% of the total assemblage.

**Table 3:** Contribution of morphospecies to macrofungal assemblages in all four different habitats

Habitat	Total morphospecies	Morphospecies	Family	Contribution (%)
Peat swamp forest	61	*Inocybe* sp.	Inocybaceae	25.11
		*Marasmius rotalis*	Marasmiaceae	18.20
		*Mycena* sp. 1	Mycenaceae	11.95
		*Marasmiellus* sp.	Marasmiaceae	9.40
		*Bjerkandera adusta*	Meruliaceae	6.72
		*Marasmius* sp. 1	Marasmiaceae	5.71
		*Trametes* sp. 3	Polyporaceae	3.36
		*Coriolopsis* sp. 1	Polyporaceae	2.46
		*Tetrapygros nigripes*	Marasmiaceae	2.31
		*Trametes* sp. 2	Polyporaceae	1.33
		*Auricularia* sp.	Auriculariaceae	1.18
		*Trametes* sp. 1	Polyporaceae	1.16
		*Dichomitus* sp.	Polyporaceae	1.14
				90.04%
Large‐scale plantation	21	*Schizophyllum commune*	Schizophyllaceae	84.30
		*Marasmiellus* sp.	Marasmiaceae	8.37
				92.66%
Monoculture smallholding	21	*Schizophyllum commune*	Schizophyllaceae	63.96
		*Ganoderma* sp. 3	Ganodermataceae	13.95
		*Marasmiellus* sp.	Marasmiaceae	7.33
		*Entoloma* sp.	Entolomataceae	2.85
		*Mycena* sp. 6	Mycenaceae	2.67
				90.75%
Polyculture smallholding	24	*Ganoderma* sp. 3	Ganodermataceae	31.20
		*Schizophyllum commune*	Schizophyllaceae	28.88
		*Entoloma* sp.	Entolomataceae	17.77
		*Marasmiellus* sp.	Marasmiaceae	11.01
		*Trametes* sp. 1	Polyporaceae	3.84
				92.71%

*Schizophyllum commune* (Figure 7e) was found in almost all sampling units at large‐scale plantation, monoculture, and polyculture smallholding, mainly on oil palm fronds. *Schizophyllum commune* was leading macrofungal morphospecies in two habitats: large‐scale plantation and monoculture smallholding. In polyculture smallholdings, surprisingly we found that *Ganoderma* sp. (Figure 7) contributed 31.20% of total assemblage morphospecies in the habitat.

## 4 DISCUSSION

This study compares the macrofungal biodiversity in four different habitats, namely logged peat swamp forest, large‐scale plantation, monoculture, and polyculture smallholdings. Our study revealed that logged peat swamp forest supports higher abundance of macrofungal clusters and more diverse morphospecies. The current results have to be viewed as conservative because not all morphospecies were collected in the plots. However, the results between habitats are comparable. A more accurate sampling, for example, through the application of molecular techniques, would result in better estimation.

### 4.1 Macrofungal morphospecies richness and abundance

Based on ANOVA analysis, we found that logged peat swamp forest contains higher macrofungal clusters and more diverse morphospecies than large‐scale oil palm plantation, monoculture, and polyculture smallholdings. This indicates that logged peat swamp forest supports more diverse macrofungal biodiversity, which might be due to environmental factors that determine the morphospecies and number of macrofungal cluster produced (López‐Quintero et al., 2012). However, between the cultivated habitats themselves, the macrofungal morphospecies and number of macrofungal cluster do not differ significantly. The patterns in the spatial distribution of fungal species hereby provide important clues about the underlying mechanisms that structure ecological communities and these are central for setting conservation priorities (López‐Quintero et al., 2012; Mueller & Schmit, 2007).

Brown et al. (2006) reported that forest patches had the highest sporocarp abundance and the greatest morphospecies richness per sample area. In contrast, coffee plantations had the lowest (Brown et al., 2006). Nevertheless, coffee plantation samples were more diverse for a given number of sporocarps than a sample of a similar size from forest patches (Brown et al., 2006). Morphospecies richness and the number of macrofungal clusters might be attributed to vegetation structure characteristics; canopy cover and canopy closure, substrate availability, and environmental factors. This is due to the changes in taxonomic and chemical composition of plant diversity which influence macrofungal productivity (Swift, Heal, & Anderson, 1979). Shifts in biological communities are expected during forest conversion because land preparation activities can cause extensive changes to the original vegetation characteristics, soil moisture retention, and other parameters (Gómez‐Hernández & Williams‐Linera, 2011; Paz et al., 2015).

Although we did not detect a significant difference in the macrofungal abundance and richness between oil palm management systems, we did find a higher richness and abundance in polyculture smallholdings compared to large‐scale plantations and monoculture smallholdings. A possible explanation is that polyculture smallholdings exhibit a higher level of habitat heterogeneity created by crop diversity (Azhar et al., 2013, 2015). Habitat heterogeneity provides suitable microclimate which influences macrofungal productivity (Gómez‐Hernández & Williams‐Linera, 2011) especially in tropical countries (Lodge, 1997). Besides that, the production of macrofungal morphospecies abundance and richness in plantation areas might also be influenced by intensive use of pesticides and fungicides (Paz et al., 2015).

### 4.2 Important environmental factors for macrofungi

Among all parameters, vegetation structure plays a vital role in the production of macrofungal communities (Bonet, Fischer, & Colinas, 2004; Calado, Lauro, & Santos‐Silva, 2009; Laganá, Loppi, & De Dominicis, 1999; Moreau & Courtecuisse, 2003; Senn‐Irlet & Bieri, 1999; Villeneuve, Grandtner, & Fortin, 1989). We found that peat swamp forest was characterized by higher percentage of canopy cover and denser canopy closure than the plantation habitat. This is due to the presence of various tree species such as *Macaranga pruinosa*,* Shorea platycarpa*, and other buttress standing trees (Sasidhran et al., 2016; Yule & Gomez, 2008). Unlike the logged peat swamp forest, our findings show a significantly lower percentage of canopy closure and canopy cover in large‐scale plantations, monoculture, and polyculture smallholdings. Similarly, Brown et al. (2006) suggested that monocultural plantations have a more open canopy than traditional farmlands and forested areas. Hence, the plantations are characterized by higher light levels, higher temperatures, and lower humidity which may have resulted in low findings of sporocarp production (Brown et al., 2006).

Canopy cover is important in shaping macrofungi diversity and productivity (Villeneuve et al., 1989). Closed canopy cover provides suitable environmental condition for macrofungal production because it reduces temperature and increases relative humidity for fungal growth (Belsky, 1994; Belsky et al., 1989). Due to humid microclimate, the soil moisture is higher in peat swamp forest than converted agriculture land. However, fungi are an adaptable species; they are able to withstand stress and adjust themselves to adapt to the most oligotrophic environments (Bergero, Girlanda, Varese, Intilli, & Luppi, 1999; Dighton, 2003; Wainwright, Al‐Wajeeh, & Grayston, 1997).

We found a greater diversity of substrates available in logged peat swamp forest compared to oil palm plantation habitats. This was probably the result of a more diverse plant community within peat swamp forest. In oil palm plantations, many fungi were found on decaying wood and oil palm bracts. Polyculture smallholdings contained a significantly greater diversity of substrates than large‐scale plantations and monoculture smallholdings. This is probably because deadwood is rarely removed in polyculture smallholdings. Previous studies have reported that saprotrophic fungi are more strongly associated with substrate availability than canopy cover (Robert, Ceska, Kroeger, & Kendrick, 2004; Santos‐Silva, Gonçalves, & Louro, 2011), and substrates are known to be important in maintaining a diverse community of fungi (Bader, Jansson, & Jonsson, 1995; Brazee et al., 2014; Heilmann‐Clausen & Christensen, 2003; Nordén, Ryberg, Götmark, & Olausson, 2004). For example, results from a study in Sarawak, Malaysia found that fungal species density increased with increasing number of substrates (such as coarse woody debris; Yamashita et al., 2008).

### 4.3 Macrofungal morphospecies composition

Our results indicate that peat swamp forest and oil palm plantation supported different macrofungal communities. Macrofungal community in the logged peat swamp forest was diverse compared to oil palm production areas. Our findings are consistent with studies carried out in Western Ghats, India (Brown et al., 2006), south‐eastern Australia (Kasel et al., 2008), and southern Brazil (Paz et al., 2015), which found that converted land does not support the original macrofungal composition of peat swamp forests.

There was no significant difference in macrofungal composition between monoculture and polyculture smallholdings. This implies that smallholdings show a similar macrofungal composition. Our SIMPER analysis shows that *Schizophyllum commune* was found in all three oil palm habitats and was the most abundant species in large‐scale oil palm plantation and monoculture smallholdings. A study in Southwestern Nigeria also found that *S. commune* is the most dominant macrofungi in rubber plantations (Osemwegie & Okhuoya, 2011). *S. commune* is not only an edible mushroom with medicinal value but is also reported to contain etiological agents (Saha et al., 2013).

*Ganoderma boninense* was the most common species in polyculture smallholdings. This could be due to the polyculture smallholdings management practices. Ganoderma are most commonly found on the remains of coconut trunks which were left on the site to rot. Retaining dead trees can potentially harbor diseases such as white rot fungus, *G. boninense* (Hushiarian et al., 2013). Antagonistic fungi, applying chemical treatments, and planting legume cover crops have been used to control *G. boninense* (Hushiarian et al.,^2013^).

## 5 CONCLUSIONS

To our knowledge, our study has provided the first empirical evidence that modification in peat swamp forest to agricultural plantation leads to definite changes in the macrofungal biodiversity. Macrofungal biodiversity was reduced when the peat swamp forest was converted into oil palm plantations due to changes in environmental factors driven by vegetation structure modification and substrate availability. Available data also indicate logged peat swamp forests are essential to the persistence of macrofungal biodiversity in tropical human‐modified landscapes. Further expansion of oil palm plantation on forest land should be prohibited in palm oil producing countries. The sustainable management of the peat swamps forest requires retaining diverse substrates such as dead wood of all sizes, species, and decay stages to maintain wood‐inhabiting fungi diversity (Gates, Mohammed, Wardlaw, Ratkowsky, & Davidson, 2011). As oil palm expansion is inevitable in the tropics, palm oil stakeholders should be encouraged to use management practices that can enhance this biodiversity (Fischer et al., 2008; Foster et al., 2011).

## CONFLICT OF INTEREST

None declared.
